# Editorial: Blood Biomarkers of Neurodegenerative Diseases

**DOI:** 10.3389/fnmol.2022.966139

**Published:** 2022-06-30

**Authors:** Thomas K. Karikari, Nicholas J. Ashton, Henrik Zetterberg, Kaj Blennow

**Affiliations:** ^1^Department of Psychiatry and Neurochemistry, Institute of Neuroscience and Physiology, The Sahlgrenska Academy, University of Gothenburg, Gothenburg, Sweden; ^2^Department of Psychiatry, University of Pittsburgh, Pittsburgh, PA, United States; ^3^Centre for Age-Related Medicine, Stavanger University Hospital, Stavanger, Norway; ^4^King's College London, Institute of Psychiatry, Psychology & Neuroscience, Maurice Wohl Clinical Neuroscience Institute, London, United Kingdom; ^5^National Institute for Health and Care Research (NIHR) Biomedical Research Centre for Mental Health & Biomedical Research Unit for Dementia at South London & Maudsley National Health Service (NHS) Foundation, London, United Kingdom; ^6^Clinical Neurochemistry Laboratory, Sahlgrenska University Hospital, Mölndal, Sweden; ^7^Department of Neurodegenerative Disease, University College London (UCL) Institute of Neurology, London, United Kingdom; ^8^UK Dementia Research Institute at University College London (UCL), London, United Kingdom; ^9^Hong Kong Center for Neurodegenerative Diseases, Hong Kong, China

**Keywords:** blood biomarker, Alzheimer's disease, tau, amyloid, neurofilament

We are rapidly entering an era where the use of easily accessible blood tests give meaningful information about the pathophysiological basis of neurodegenerative diseases. Recent technical and methodological advances, aided by the rise of well-characterized research cohorts, have propelled the neurodegeneration field in a direction toward several important and sought-after applications of blood biomarkers; as screening or risk assessment tools, or as therapy monitoring in clinical trials. The key candidates include biomarkers fulfilling the AT(N) criteria for the definition and staging of Alzheimer's disease (AD) (Ashton et al., 2020; Teunissen et al., 2021; Karikari et al., 2022). However, the widespread applications of these biomarkers would require prior verification in diverse populations. Moreover, there is a need to characterize demographic, lifestyle and medical conditions that can affect the production and clearance of these blood biomarkers. Furthermore, blood biomarkers of other key molecular processes involved in AD (e.g., inflammation) and, importantly, other neurodegenerative diseases (e.g., TDP-43 and α-synuclein) are actively being explored to improve disease identification, characterization, and differential diagnosis.

We proposed a Research Topic focused on gathering further evidence on the aforementioned topics. Forty-five manuscripts were submitted for consideration and 25 of these were accepted for publication following the peer-review process. The accepted manuscripts are summarized below, divided into seven broad areas.

## Alzheimer's, Parkinson's and Other Neurodegenerative Diseases

In a study of 277 participants, Jiao et al. reported significant increases in plasma total-tau and NfL in AD vs. controls. A diagnostic model incorporating these blood biomarkers and age, sex, and *APOE* allele gave a diagnostic accuracy of 89%, sensitivity of 82% and specificity of 84%. In evaluating 463 AD patients and 1,389 controls matched according to age, sex and body mass index, Chen X. et al. found significantly higher levels of serum cystatin C in AD vs. control participants. Serum cystatin C levels did associate with cognitive function and the risk of cognitive impairment. In an investigation that combined experimental and bioinformatic approaches, Sabaie et al. showed that the *BCAS4/miR-185-5p/SHISA7* competing endogenous RNA axis in the blood and brains of people with AD.

Xu et al. sought to explore this association further by investigating relationships between heme oxygenase-1 levels, iron content and hemoglobin levels. The authors showed that in people with Parkinson's disease (PD) there were increased levels of heme oxygenase-1 and low levels of hemoglobin, which they concluded to be a putative mechanisms of iron deposition in PD. Hu et al. reported that concentrations of the anti-inflammatory neuropeptides pituitary adenylate-cyclase activating polypeptide (PACAP) and vasoactive intestinal peptide (VIP) were increased in people living with PD vs. unaffected controls. Measures of mood disorder and non-motor symptoms were associated with serum VIP and PACAP levels, respectively. Zhang W. et al. integrated experimental and computational methods to show that expression of the signal sequence receptor subunit 1 gene was significantly upregulated in the blood of animal models of PD.

Vacchiano et al. showed significant correlations between plasma and cerebrospinal fluid (CSF) levels of NfL in paired samples, and both showed high performances to difference ALS patients from ALS mimics despite CSF NfL being slightly superior (AUC = 92%) to plasma NfL (AUC = 87%). High concentrations of NfL in either CSF or plasma signified faster disease progression and poorer longitudinal survival.

In a publication on Huntingtin disease, Ingannato et al. showed that individuals with bulbar onset disease had higher plasma NfL levels and later age of onset compared with the spinal onset variant.

An original report by Ye et al. indicated that serum levels of cystatin C were associated with measures of disability in patients with multiple systems atrophy including those with predominant cerebellar ataxia (MSA-C) but not those with predominant Parkinsonism. MSA-C patients with low vs. high levels of serum cystatin C demonstrated a difference in the probability of survival over a 5-year duration. Guan et al. showed that serum uric acid levels were significantly decreased in people diagnosed with Meige's syndrome vs. controls and predicted severe clinical symptoms.

## Mild Cognitive Impairment

Liang et al. reported that interleukin-2, an immunoregulatory protein, is a potentially better blood biomarker for cognitive decline in amnestic mild cognitive decline than each of plasma Aβ42/Aβ40, total-tau and p-tau181. In a 9-year longitudinal study, Chen C. et al. reported that older adults with high uric acid levels in blood have reduced risk of developing mild cognitive impairment. Ngwa et al. described genome-wide DNA methylation patterns associated with aerobic exercise in people with mild cognitive impairment.

## Normal Aging

Zecca et al. studied age effects on plasma Aβ42 levels, reporting a significant positive correlation in the whole cohort. When separated into age groups, they found a significant inverse association of plasma Aβ42 with age in individuals <35 years old, a significant positive correlation in those aged 35–65 years old, and no correlation in those over 65 years.

Orihashi et al. showed that soluble triggering receptor expressed on myeloid cells 2 (sTREM2) levels did not correlate with baseline or longitudinal changes in the volumes of specific brain regions closely linked with cognitive function in normal aging. In a report by Kapoor et al., the levels of endothelial progenitor cells and vascular endothelial growth factor D was each associated with small vessel disease after accounting for age and sex. In a study performed in an APP/PS1 mouse model, Zhang L. et al. explored dynamic changes in Aβ42 aggregates in peripheral vs. brain-derived sources. Using immunoassays that differentiate monomeric from oligomeric and total Aβ42 species, the authors showed that the levels of Aβ42 monomers and oligomers were significantly decreased and increased, respectively, in 9- vs. 3-month-old animals. Moreover, intestinal levels of Aβ42 were also higher at 9- vs. 3-months.

## Kidney Disease and Renal Function

Hou et al. investigated the utility of plasma biomarkers in end-stage renal disease. They reported marginally higher levels of the plasma NfL in patients with vs. without cognitive impairment but the levels did not associate with cognitive function or biochemical parameters. However, the levels of plasma Aβ40, Aβ42 and total-tau were not different between end-stage renal disease participants with or without cognitive decline.

## Analytical Methods

Chatterton et al. reported a proof-of-concept study to detect cell-free DNA in blood plasma by combining bisulfite sequencing and computational processing approaches.

Chang et al. examined preanalytical handling protocols for the assessment of alpha-synuclein and NfL in plasma. The results showed that K_2_-EDTA and K_3_-EDTA tubes had equivalent performances for assessing both biomarkers. However, sample centrifugation at 4 °C led to lower and less reproducible levels of plasma α-synuclein measured.

## Genome-Wide Association Studies

In a genome-wide association study, Chen H. et al. identified over 160 metabolites that did associate significantly with several neurodegenerative diseases. For instance, X-11529 and X-13429 was associated with frontotemporal dementia while 2-methoxyacetaminophen sulfate was associated with amyotrophic lateral sclerosis. Furthermore, Zhao et al. reported suggestive associations of interleukin-16 and macrophage inflammatory protein-1 beta with PD risk, but age of onset did not associate with inflammatory cytokine profiles.

## Review Articles

Asadi et al. reviewed publications on the value of stress granules in the neurodegenerative diseases and the proteins that these granules consist of. Guo et al. presented a narrative review of recent advances in peptide- and peptoid-based biomarker detection assays and technology platforms used in the AD field.

## Conclusion

The articles published in this Research Topic cover a broad range of topics of direct relevance to the pathogenesis and etiology of neurodegenerative diseases. Other articles covered preanalytical handling and clinical performances of blood biomarkers, as well as lifestyle and age-related factors that can affect their performances.

## Author Contributions

TK prepared an initial draft of this editorial that was carefully revised by NA, HZ, and KB. All authors approved the final version for submission.

## Funding

TK was funded by the Swedish Research Council (Vetenskapsrådet #2021-03244), the Alzheimer's Association Research Fellowship (#AARF-21-850325), the BrightFocus Foundation (#A2020812F), the Emil and Wera Cornells foundation, the International Society for Neurochemistry's Career Development Grant, the Swedish Alzheimer Foundation (Alzheimerfonden; #AF-930627), the Swedish Brain Foundation (Hjärnfonden; #FO2020-0240), the Swedish Dementia Foundation (Demensförbundet), the Swedish Parkinson Foundation (Parkinsonfonden), Gamla Tjänarinnor Foundation, the Aina (Ann) Wallströms and Mary-Ann Sjöbloms Foundation, the Agneta Prytz-Folkes & Gösta Folkes Foundation (#2020-00124), the Gun and Bertil Stohnes Foundation, and the Anna Lisa and Brother Björnsson's Foundation. NA was supported by the Swedish Alzheimer Foundation (Alzheimerfonden; #AF-931009), Hjärnfonden and the Swedish Dementia Foundation (Demensförbundet). HZ is a Wallenberg Scholar supported by grants from the Swedish Research Council (#2018-02532), the European Research Council (#681712), Swedish State Support for Clinical Research (#ALFGBG-720931), the Alzheimer Drug Discovery Foundation (ADDF), USA (#201809-2016862), the AD Strategic Fund and the Alzheimer's Association (#ADSF-21-831376-C, #ADSF-21-831381-C, and #ADSF-21-831377-C), the Olav Thon Foundation, the Erling-Persson Family Foundation, Stiftelsen för Gamla Tjänarinnor, Hjärnfonden, Sweden (#FO2019-0228), the European Union's Horizon 2020 research and innovation programme under the Marie Skłodowska-Curie grant agreement No 860197 (MIRIADE), European Union Joint Program for Neurodegenerative Disorders (JPND2021-00694), and the UK Dementia Research Institute at UCL.KB is supported by the Swedish Research Council (#2017-00915), the Alzheimer Drug Discovery Foundation (ADDF), USA (#RDAPB-201809-2016615), the Swedish Alzheimer Foundation (#AF-742881), Hjärnfonden, Sweden (#FO2017-0243), the Swedish state under the agreement between the Swedish government and the County Councils, the ALF-agreement (#ALFGBG-715986), and European Union Joint Program for Neurodegenerative Disorders (JPND2019-466-236). The funders had no role in data collection, analysis or decision to publish.

## Conflict of Interest

HZ has served at scientific advisory boards and/or as a consultant for Abbvie, Alector, ALZPath, Annexon, Apellis, Artery Therapeutics, AZTherapies, CogRx, Denali, Eisai, Nervgen, Novo Nordisk, Pinteon Therapeutics, Red Abbey Labs, reMYND, Passage Bio, Roche, Samumed, Siemens Healthineers, Triplet Therapeutics, and Wave, and has given lectures in symposia sponsored by Cellectricon, Fujirebio, Alzecure, Biogen, and Roche. KB has served as a consultant, at advisory boards, or at data monitoring committees for Abcam, Axon, BioArctic, Biogen, JOMDD/Shimadzu. Julius Clinical, Lilly, MagQu, Novartis, Ono Pharma, Pharmatrophix, Prothena, Roche Diagnostics, and Siemens Healthineers, HZ and KB are co-founders of Brain Biomarker Solutions in Gothenburg AB, a GU Ventures-based platform company at the University of Gothenburg. The remaining authors declare that the research was conducted in the absence of any commercial or financial relationships that could be construed as a potential conflict of interest.

## Publisher's Note

All claims expressed in this article are solely those of the authors and do not necessarily represent those of their affiliated organizations, or those of the publisher, the editors and the reviewers. Any product that may be evaluated in this article, or claim that may be made by its manufacturer, is not guaranteed or endorsed by the publisher.
